# Survival of Enterohemorrhagic *Escherichia coli* O104:H4 Strain C227/11Φcu in Agricultural Soils Depends on *rpoS* and Environmental Factors

**DOI:** 10.3390/pathogens10111443

**Published:** 2021-11-05

**Authors:** Katharina Detert, Herbert Schmidt

**Affiliations:** Department of Food Microbiology and Hygiene, Institute of Food Science and Biotechnology, University of Hohenheim, Garbenstraße 28, 70599 Stuttgart, Germany; Katharina.detert@uni-hohenheim.de

**Keywords:** EHEC/EAEC, O104:H4, C227/11Φcu, survival, agricultural soil, *rpoS*, *fliC*

## Abstract

The consumption of contaminated fresh produce caused outbreaks of enterohemorrhagic (EHEC) *Escherichia coli*. Agricultural soil might be a reservoir for EHEC strains and represent a contamination source for edible plants. Furthermore, the application of manure as fertilizer is an important contamination route. Thus, the German fertilizer ordinance prohibits the use of manure 12 weeks before crop harvest to avoid pathogen transmission into the food chain. In this study, the survival of *E. coli* O104:H4 strain C227/11Φcu in soil microenvironments with either diluvial sand or alluvial loam at two temperatures was investigated for more than 12 weeks. It was analyzed whether the addition of cattle manure extends EHEC survival in these microenvironments. The experiments were additionally performed with isogenic Δ*rpoS* and Δ*fliC* deletion mutants of C227/11Φcu. The survival of C227/11Φcu was highest at 4 °C, whereas the soil type had a minor influence. The addition of cattle manure increased the survival at 22 °C. Deletion of *rpoS* significantly decreased the survival period under all cultivation conditions, whereas *fliC* deletion did not have any influence. The results of our study demonstrate that EHEC C227/11Φcu is able to survive for more than 12 weeks in soil microenvironments and that RpoS is an important determinant for survival.

## 1. Introduction

Enterohemorrhagic *Escherichia coli* (EHEC) strains can cause diarrhea, hemorrhagic colitis and hemolytic-uremic syndrome (HUS) in humans and have been associated with many serious food-borne outbreaks [1,2,3,4,5]. Cattle are considered to be the main reservoir for EHEC, and several outbreaks were caused by the consumption of animal-derived foods [3,5]. In recent years, a number of outbreaks caused by classical EHEC, and hybrid enteroaggregative/enterohemorrhagic *E. coli* (EHEC/EAEC) were attributed to the consumption of non-heated vegetables [4,6,7]. Since fresh produce is mostly consumed raw, contamination of these foods may represent a serious health risk for consumers. In 2011, a novel hybrid EHEC/EAEC strain of serotype O104:H4 led to a large outbreak of diarrhea and HUS in Germany. Fenugreek sprouts were identified as the most likely vehicle of infection [8,9,10]. As a response to increasing infection sources of non-animal origin, and based on laboratory experiments, it has been investigated whether pathogens such as EHEC are able to colonize plants as secondary hosts [11,12,13,14].

The transmission of bacterial pathogens can occur from contamination of environmental, animal, or human sources and can occur along the whole food chain [15,16]. Thereby, one important contamination source is agricultural soil [16]. Contamination of soil with pathogens can occur by irrigation with contaminated water or due to the use of organic fertilizer [11,16]. Especially the application of cattle manure for soil fertilization has been recognized as an important route through which EHEC can contaminate plants and vegetables [17,18,19]. Pathogens present in agricultural soils have the potential to be transported to surface or groundwater [20]. Further possibilities are the attachment to plant surfaces and subsequent internalization into plant tissues that present a serious risk to public health [12,21,22]. To avoid pathogen transmission into the food chain, the German fertilizer ordinance prohibits the use of manure 12 weeks before crop harvest. However, different studies have shown that human pathogens survive longer than 12 weeks in the soil and thus continue to pose a risk for human infection [23,24]. The survival of *Salmonella enterica*, *Vibrio cholerae*, *Campylobacter jejuni* and pathogenic *E. coli* in soils has been investigated in different studies [20,23,24,25,26,27,28]. It was shown that survival in soil depends on various biotic factors such as the surrounding prokaryotic microbiota and abiotic factors such as nutrient availability, pH, moisture, and temperature [29]. In contrast, only little is known about the influence of bacterial determinants that are essential for soil survival. The identification of such factors is essential to better understand the survival ability in the soil environment. This is of particular interest since pathogenic *E. coli* strains were able to colonize plants when grown in contaminated soil [13,14,30,31,32].

In this study, we investigated the survival of *E. coli* O104:H4 strain C227/11Φcu [33] in defined agricultural soil microenvironments depending on different soil types and different conditions on a laboratory scale. We used two different agricultural soil types, the nutrient-poor diluvial sand (DS), which is an arenic-luvisol with minor amounts of silty sand and 5.5% clay, and the more nutritious alluvial loam (AL), which is a gleyic-fluvisol with heavy sandy loam and 27.5% clay [34,35]. Both soil types were well described by Schreiter et al. [35] and have been used in previous studies [36,37,38,39]. Additionally, we used two temperatures (4 °C and 22 °C), which mirrored environmental conditions. Furthermore, cattle manure was added to the soil samples since we hypothesized that soil fertilization by manure application even increase the survival ability of EHEC in soil. In addition, we investigated the impact of RpoS and FliC, two bacterial factors that were hypothesized to influence the survival of *E. coli* O104:H4 strain C227/11Φcu. Therefore, we constructed isogenic Δ*rpoS* and Δ*fliC* deletion mutants of C227/11Φcu. The sigma factor RpoS represents the major factor involved in the general stress response in *E. coli* [40,41,42]. Since a variety of stress conditions may be present in soil, such as nutrient deficiency, we assumed that *rpoS* deletion mutants might be restricted in survival in the soil environment. Some data have already been published about the influence of bacterial determinants on survival in soil. Van Hoek et al. [43] analyzed the role of RpoS for the long-term survival of *E. coli* O157 and concluded that further deletion and complementation studies are required. We further hypothesized that the deletion of the flagellin gene *fliC* might influence bacterial survival in soil. The bacterial fitness in soil is enhanced by adhesion to soil particles and biofilm formation [44,45]. Flagella are primarily involved in cell motility, but they also participate in cellular processes such as adhesion or biofilm formation [44,45,46,47]. To analyze the survival of *E. coli* O104:H4 strain C227/11Φcu and its isogenic Δ*rpoS* and Δ*fliC* deletion mutants in soil, we established a method for the inoculation of soil microenvironments and used cultivation-dependent methods for analysis.

## 2. Results

### 2.1. Construction of Deletion Mutants

To investigate the influence of FliC and RpoS on the survival of EHEC in soil, isogenic Δ*fliC* and Δ*rpoS* mutants of *E. coli* O104:H4 strain C227/11Φcu were constructed. Consequently, C227/11Φcu was transformed with the modified plasmid pKEC1.5 as described in 4.3. Following the transformation of pCP20, which encodes an FLP recombinase, the resistance gene was excised. The resulting deletion mutants were verified by PCR using primers that were specific for the corresponding genetic background (Figures S1–S3). Additionally, DNA sequence analysis was performed as described to confirm the correct generation of *E. coli* O104:H4 C227/11Φcu Δ*rpoS* and *E. coli* O104:H4 C227/11Φcu Δ*fliC* (Table 1). All deletion mutants were complemented with plasmids as described.

### 2.2. Transcription of Cloned fliC or rpoS Genes on Complementation Plasmids

After sequence analysis, which confirmed the correct insertion of deleted genes in the complementation plasmid, the expression of cloned *fliC* and *rpoS* genes in the recombinant plasmids was analyzed. Therefore, C227/11Φcu, C227/11Φcu Δ*rpoS*, C227/11Φcu Δ*fliC*, and mutant strains carrying the complementation plasmids were grown under standard batch conditions, and the total RNA was isolated. PCR analysis of the *rpoS* and *fliC* cDNA with specific primers (Table 2) and subsequent agarose gel electrophoresis demonstrated the expected PCR products of 993 bp for *rpoS* and 1050 bp for *fliC* for the wild type and the complemented deletion mutants (Figure S4). A reverse transcriptase negative control was applied to avoid false-positive results. Amplification of the cDNA prepared from the complemented genes, as well as from the deletion mutants, was negative (Figure S4).

### 2.3. Survival of E. coli O104:H4 Strain C227/11Φcu in Different Soil Types under Different Temperatures

**Figure 1:**
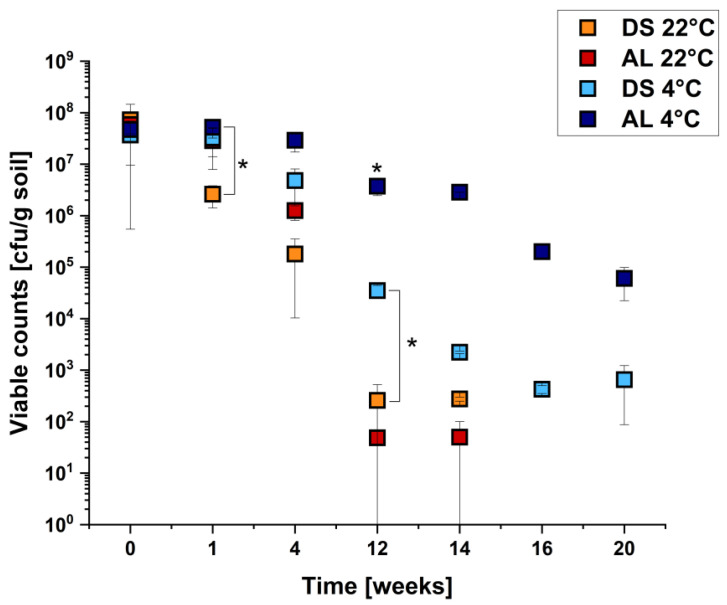
Analysis of survival of *E. coli* O104:H4 C227/11Φcu in soil microenvironments depending on soil type and temperature (as indicated). The soil was inoculated with 10^8^ cfu/g soil and incubated for several weeks. Data are means ± standard errors of the experiments performed in triplicates. * Values are statistically significant.

The survival of pathogenic *E. coli* in the agricultural soil microenvironment was investigated using different soil types and temperatures. Therefore, 10^8^ cfu/g of strain C227/11Φcu were inoculated into diluvial sand (DS) and alluvial loam (AL) microenvironments. The samples were incubated at either 4 °C or 22 °C for up to 20 weeks. The total aerobic viable counts were monitored during the entire cultivation period. Since no bacterial colonies were detected on the TBX control plates at all time points, all colonies on TBX agar were considered as C227/11Φcu. The viable counts of all four temperature/soil combinations are shown in Figure 1. The survival of C227/11Φcu in AL and DS was additionally analyzed using the software GInaFiT and the Weibull model (Table 3 and Figure S5). The application of the Weibull model for the survival curves of *E. coli* O104:H4 C227/11Φcu in soil resulted in good fits with an average root mean square error of 0.5647 ± 0.18 and an average regression coefficient (R^2^) of 0.9654 ± 0.0015. In addition, it was possible to determine the times required for the first decimal reduction and the time until the detection limit of 10^2^ cfu/g soil is reached. The resulting model curves are shown in Figure S5A–D, and the obtained and calculated parameters are provided in Table 3.

The strongest decrease in viable counts was found for C227/11Φcu in AL and DS incubated at 22 °C with a total reduction of approximately 5 log units each within 12 weeks (Figure 1). During the first 4 weeks, more bacteria survived in AL than in DS, but these differences balanced out after 12 weeks at 22 °C. After 16 weeks, no cultivable bacteria were detected at 22 °C in both soils. According to the Weibull modulation, the detection limit was reached for DS and AL after ±13.3 and ±12.3, respectively (Table 3). The situation was different at 4 °C. The cfu/g in both soils were nearly similar in the first 4 weeks of incubation, whereas a stronger decrease in viable counts was observed between weeks 4 and 12 in DS. After 20 weeks, viable counts of about 10^3^ cfu/g were detected. The incubation of C227/11Φcu in AL for 20 weeks at 4 °C resulted in a slight decrease in cfu/g from 10^8^ to 10^5^ cfu/g soil. The best survival of C227/11Φcu was found for AL at 4 °C. The influence of temperature and soil type was further highlighted with the application of the Weibull model. The first decimal reduction for the other three temperature/soil combinations occurred after ~1.15 to 2.89 weeks (Table 3). In comparison, the first decimal reduction was achieved after ~11 weeks, and no 4 log reduction was found for C227/11Φcu in AL at 4 °C during the incubation period of 20 weeks.

Summarizing the results, it was clearly shown that under all conditions applied, strain C227/11Φcu could survive for at least 12 weeks in soil microenvironments with a maximum decrease of 10^6^ cfu/g at 22 °C in AL and a minimum decrease of 10^1^ cfu/g in AL at 4 °C. Survival up to 20 weeks occurred in DS and AL at 4 °C, with a decrease in cfu/g of 10^5^ and 10^3^, respectively. 

### 2.4. Addition of Cattle Manure to Soil Microenvironments Improves Survival of E. coli O104:H4 Strain C227/11Φcu

**Figure 2:**
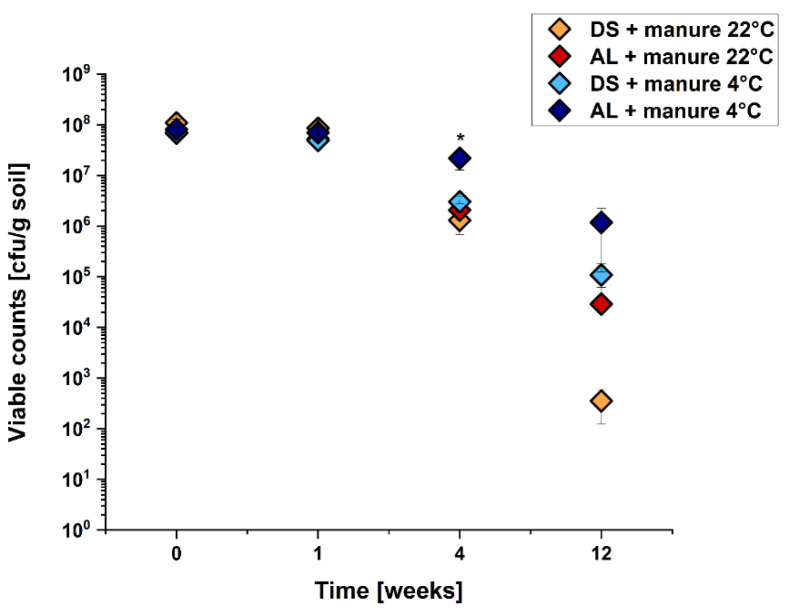
Analysis of soil survival of *E. coli* O104:H4 C227/11Φcu depending on soil type, temperature and cattle manure addition (as indicated). The soil was treated with manure and inoculated with 10^8^ cfu/g soil and incubated for several weeks. Data are means ± standard errors of the experiments performed in triplicates. * Value of AL + manure at 4 °C is statistically significant with regard to the other conditions at week 4.

In a further experimental set-up, we wanted to investigate whether the application of cattle manure influences the survival of *E. coli* O104:H4 strain C227/11Φcu in the soil microenvironments. The manure was mixed with the soil prior to inoculation, and the quantity was calculated based on the maximum amount of nitrogen allowed in agricultural practices (170 kg N/ha). The viable counts of all four temperature/soil combinations are shown in Figure 2. 

They show that the application of cattle manure increased the survival of *E. coli* O104:H4 strain C227/11Φcu when the samples were stored at 22 °C. In contrast to the prior experiments shown in Figure 1, no decrease in viable counts of the 22 °C samples was detected within the first week of incubation. During the further incubation period, the viable counts decreased. However, the rate of decrease was lower compared to those depicted in Figure 1, indicating a positive effect of manure on the survival of C227/11Φcu. The incubation in AL at 22 °C without cattle manure resulted in a decrease of 5 log units within 12 weeks (Figure 1), which was only 3 log units when cattle manure was added (Figure 2). 

Taken together, it was shown that the addition of cattle manure improved the survival of C227/11Φcu in DS and AL samples stored at 22 °C, but not at 4 °C.

### 2.5. Influence of RpoS and FliC on the Survival of E. coli O104:H4 Strain C227/11Φcu in Soil Samples

**Figure 3:**
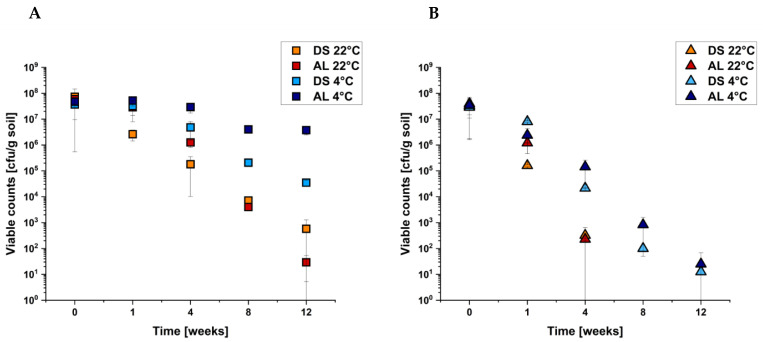
Analysis of soil survival of *E. coli* O104:H4 C227/11Φcu (**A**) and its isogenic Δ*rpoS* deletion mutant (**B**) depending on soil type and temperature (as indicated). The soil was inoculated with 10^8^ cfu/g soil and incubated for 12 weeks. Data are means ± standard errors of the experiments performed in triplicates. (**A**) is taken from Figure 1 as a comparison.

In the next step, we investigated the impact of the sigma factor gene *rpoS* and the flagellin gene *fliC*, using the respective isogenic deletion mutants for soil experiments. We hypothesized that RpoS and FliC play an important role in soil survival. First, *E. coli* O104:H4 strain C227/11Φcu and its Δ*rpoS* deletion mutant were used to inoculate the soil samples as described above. The detected viable counts of all four temperature/soil combinations are shown in Figure 3A,B. 

The deletion of *rpoS* significantly decreased the survival under all cultivation conditions that become apparent through the fast decrease in viable counts in all samples. For AL and DS at 4 °C, a decrease in viable counts from 10^8^ to 10^1^ CFU/g soil was detected within 12 weeks (Figure 3B), which demonstrates the highest survival ability. In contrast, no cultivable bacteria were detectable in both soil microenvironments with a detection limit of 10 cfu/g soil after 8 weeks of incubation at 22 °C. Compared to the deletion mutant, the respective complemented deletion mutant showed higher survival in all four samples. The difference was observed, especially within the first 4 weeks (Figure S6A). The wild-type level was not completely achieved, but the differences between the deletion mutant and the complemented strain confirmed that the sigma factor RpoS is an important determinant for the survival of *E. coli* O104:H4 strain C227/11Φcu in soil under the conditions applied. 

**Figure 4:**
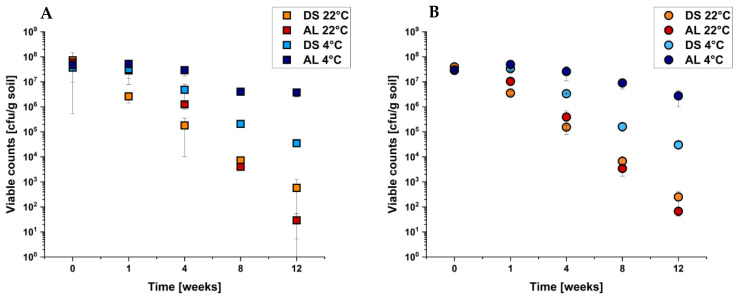
Analysis of soil survival of *E. coli* O104:H4 C227/11Φcu (**A**) and its isogenic Δ*fliC* deletion mutant (**B**) depending on soil type and temperature (as indicated). The soil was inoculated with 10^8^ cfu/g soil and incubated for 12 weeks. Data are means ± standard errors of the experiments performed in triplicates. (**A**) is taken from Figure 1 as a comparison.

The same experiments were performed using C227/11Φcu and its isogenic Δ*fliC* deletion mutant. The results of all four temperature/soil combinations are shown in Figure 4A,B. 

Here, we did not find any differences in soil survival between C227/11Φcu (Figure 4A), the deletion mutant (Figure 4B) and the complemented deletion mutant C227/11Φcu Δ*fliC*/pFJ03 (Figure S6B). The reduction in viable counts during the incubation time was identical for all tested conditions. 

The obtained results showed that the sigma factor RpoS is an important determinant for soil survival of C227/11Φcu. The survival in soil was significantly reduced in the respective Δ*rpoS* deletion mutant. In contrast, the flagellin gene *fliC* seems not to be essential for the survival of the tested EHEC strain in the two soil types.

## 3. Discussion

Organic fertilizer has been proposed as a potential contamination source since cattle are regarded as the primary reservoir of enterohemorrhagic *E. coli* [51,52,53]. The use of cattle manure as soil fertilizer can result in the introduction of pathogens into soil [17,19,54]. Environmental soil as a contamination source of edible plants was underestimated for a long time, but several outbreaks were already associated with contaminated soil [55,56]. The identification of factors, which influence the survival rate of EHEC, is a prerequisite to better understanding the survival ability in the soil environment. This is of particular interest as pathogenic *E. coli* strains were able to colonize plants when grown in contaminated soil [13,14,30,31,32]. 

The results of the current study have shown that the survival of EHEC O104:H4 strain C227/11Φcu is dependent on temperature, soil type and a functional RpoS in the used soil microenvironments. The EHEC strain survived for several weeks in the soil microenvironment. The decline of viable counts differed depending on the applied cultivation conditions and thus indicated the effect of soil type and temperature. Thereby, the survival depended more strongly on the low temperature than on the soil type. This was demonstrated by cultivation-dependent methods as well as by modelling with the GInaFiT software to assess microbial survival curves. The application of the Weibull model for the survival curves of *E. coli* O104:H4 C227/11Φcu in soil resulted in good fits and further highlighted the influence of temperature and soil type. The modulation was not possible for the other survival data since six observations or more are needed for modelling.

Jiang et al. [57] also observed a greater reduction in viable counts of *E. coli* O157:H7 in unautoclaved soil at 21 °C compared to 5 °C. A higher survival at lower temperatures was also found for *L. monocytogenes* and *Salmonella enterica* [58,59]. The reduction in survival ability at higher temperatures has been correlated with higher metabolic activity and thus faster utilization of the available nutrients [27,57,58,59]. Furthermore, it has been assumed that the reduced metabolic activity of the soil microbiota at lower temperatures enhances the survival of pathogens in soil [60,61]. In this study, we analyzed the survival under nearly constant temperatures. In a non-host environment such as soil, the temperature is fluctuating, which has been shown to reduce the survival of *E. coli* O157:H7 in manure amended soil [62]. 

As demonstrated in different studies, the soil type had a strong effect on the survival of *Escherichia coli*, *Listeria monocytogenes* and *Salmonella enterica* [20,27,38]. In the current study, the survival of C227/11Φcu was enhanced in AL, which might be correlated to higher carbon and nitrogen contents [34,35]. Comparable results were found for different *Salmonella enterica* strains that were inoculated into the same soil types [38]. These results strengthen the hypothesis that nutrient availability in soil is one of the main factors, which influences the survival ability of EHEC in soil. Cattle manure used as organic fertilizer can be considered a contamination source, and the application of manure can influence the survival rate of pathogens through the input of nutrients [63]. The number of pathogenic *E. coli* contained in manure is usually between 10^2^ and 10^5^ cfu/g [64,65,66]. In contrast, super-shedding animals may excrete *E. coli* O157 at levels up to >10^7^ cfu per gram of feces [19,67,68,69]. In the current study, we used high inoculation levels of 10^8^ cfu/g soil since we wanted to analyze the survival of C227/11Φcu for several weeks and did not want to fall below the detection limit. Previous studies demonstrated that *E. coli* O157:H7 was able to survive in neutral soils for 33 days while the persistence was prolonged by the addition of contaminated manure compost to nearly 200 days [23]. In the current study, the addition of nutrients in the form of cattle manure enhanced the survival of C227/11Φcu in DS and AL at 22 °C. The survival at 4 °C was not influenced by the manure application since the *E. coli* metabolism is reduced at low temperatures. 

Diluvial sand and alluvial loam also differ in clay content, water holding capacity and particle size distribution. Clay content has long been known to enhance the persistence ability of pathogens [70,71]. The survival rate of *L. monocytogenes*, for example, was lower in sandy soil compared to sandy loam soils [72]. Furthermore, the survival of *E. coli* O157:H7 was found to decrease with greater sand content in manure-amended loam soils and was improved in finer-textured, clayey soils [70]. Higher survival rates are explained by the adsorption of microorganisms onto soil particles since clay minerals influence bacterial attachment, metabolic activity, colonization, or biofilm formation [73,74,75,76]. Thereby, bacterial cells are protected against microbial predators. Bacterial association with soil particles is influenced by a range of factors, including, e.g., cell motility or the presence of extracellular polysaccharides [77,78]. Flagella are primarily involved in cell motility, but they also participate in cellular processes such as adhesion or biofilm formation [44,45,46,47]. In contrast, we did not find any effect of the Δ*fliC* deletion on the survival of *E. coli* O104:H4 strain C227/11Φcu in soil. Therefore, we assumed that the loss of motility did not affect soil survival of C227/11Φcu in the investigated soil types. In addition, adherence ability provided by flagella seems not to be important for soil survival. 

In contrast, the sigma factor RpoS was identified as an important determinant for the survival in soil. RpoS is the master regulator of the general stress response in *E. coli* and controls a large set of approximately 500 genes [40,41]. For this reason, RpoS is necessary for survival under a variety of stress conditions [42]. In the soil environment, *E. coli* is exposed to different stresses, such as various temperature conditions, osmotic stress, oxidative stress, desiccation, and nutrient starvation. Due to the induction of RpoS-controlled general stress response, *E. coli* can rapidly adapt to diverse stress conditions present in soil [40,79,80,81,82]. A study by Somorin et al. [82] showed that RpoS is highly conserved in long-term soil-persistent *E. coli* strains and that a functional RpoS is essential for the long-term survival of *E. coli* in soil. In addition, van Hoek et al. [43] also demonstrated that the long-term soil survival of *E. coli* O157:H7 for up to 200 days depended on a functional *rpoS* and that those strains carrying mutations in their *rpoS* gene were incapable of long-term survival. The authors concluded that further deletion and complementation studies are required. In the current study, we used isogenic Δ*rpoS* deletion mutants and complemented strains and found a significant reduction in survival ability. Moreover, the results of our study demonstrate that C227/11Φcu is exposed to different stresses during persistence in soil and that RpoS is essential for the soil survival of the tested EHEC strain.

## 4. Materials and Methods

### 4.1. Bacterial Strains and Plasmids

Bacterial strains and plasmids used in this study are listed in Table 1. Strains were routinely grown in LB broth (10 g/L tryptone, 10 g/L NaCl, 5 g/L yeast extract, pH 7.0) in a rotary shaker at 37 °C and 180 rpm. For the preparation of solid media, 15 g/L agar was added. If required, kanamycin (kanamycin sulfate, Roth) or chloramphenicol (Roth) were added to final concentrations of 50 μg/mL and 25 μg/mL, respectively. Plasmids were prepared from overnight cultures of the respective *E. coli* strain using a QlAprep Spin Miniprep Kit (Qiagen, Hilden, Germany) according to the manufacturer’s recommendations. 

### 4.2. Preparation of Electrocompetent Bacterial Cells and Electroporation

Electrocompetent cells were prepared as described previously with minor modifications [83]. To each cell aliquot, 300 ng of the PCR product or 30 ng plasmid DNA was added. The transformation was carried out by electroporation (25 μF, 200 Ω, 2.5 kV, 5  ±  0.2 ms) using electroporation cuvettes (2 mm; Bio-Rad) and a GenePulser Xcell electroporation system (Bio-Rad, Hercules, CA, USA).

### 4.3. Construction of Gene Deletion Mutant

Gene deletions were constructed using the lambda red recombinase system as described previously [48,49,83]. For mutagenesis of *E. coli* O104:H4 strain C227/11Φcu, plasmid pKEC1.5 was used instead of pKD46 [48]. Since *E. coli* O104:H4 is resistant against ampicillin, the TEM-1 beta-lactamase gene of plasmid pKD46 was replaced by a chloramphenicol-acetyltransferase (*cat*) gene. The primers used for mutagenesis were constructed using Serial Cloner, version 2.6.1 (SerialBasics; Franck Perez, Paris, France) and are listed in Table 2. The deletion mutants were verified by PCR and double-stranded DNA sequence analysis as described previously [84].

### 4.4. Plasmid Construction

For the construction of plasmid pFJ01, the *cat* gene, including a 375 bp upstream sequence, was amplified using pCP20 as a template. The PCR product and plasmid pBR322 were digested with the restriction endonuclease *PvuI*, and the PCR product was subsequently ligated into the backbone plasmid pBR322 and transformed into *E. coli* DH5α. PCR, restriction digestion, ligation and transformation were performed as described [37,48]. Plasmid pFJ01 was further used as a backbone plasmid for the deletion mutant complementation.

### 4.5. Complementation of Deletion Mutants

For the complementation of the deletion mutants, genomic DNA of *E. coli* O104:H4 C227/11Φcu was isolated with a DNeasy Blood and Tissue Kit (Qiagen, Hilden, Germany), according to the manufacturer’s recommendations, and used as a template. Primers were designed using Serial Cloner, version 2.6.1 (SerialBasics; Franck Perez, Paris, France). Amplification of the genes was analyzed by agarose gel (1% *w*/*v*) electrophoresis. The PCR samples were subsequently purified using a PCR Purification Kit (Qiagen, Hilden, Germany), according to the manufacturer’s recommendations (QIAquick PCR Purification Handbook 01/2020). The inserts and the constructed pFJ01 were double digested with restriction endonucleases *BamHI* and *HindIII* and purified as mentioned above. Ligation of vector and inserts was performed using a T4 DNA ligase (ThermoFisher Scientific, Waltham, MA, USA) and a vector-to-insert ratio of 1:3. The resulting plasmids pFJ02 and pFJ03 were transformed in competent *E. coli* DH5α cells as described above. Recombinant plasmids were confirmed by sequence analysis and finally transformed in the deletion mutants using electroporation as mentioned above.

### 4.6. Analysis of Gene Transcription

The expression of the genes cloned into the complementation plasmids was verified by transcription analysis as described earlier [85,86]. Deletion mutants of *E. coli* O104:H4 C227/11Φcu and the mutants harbouring complementation plasmids were grown in LB medium at 37 °C and 180 rpm until OD_600nm_ of 1.0 was reached. Next, 500 µL of the culture was transferred to 1 mL of RNAprotect bacterial reagent (Qiagen, Hilden, Germany). The RNA isolation was carried out by using the QIAGEN “RNeasy Mini Kit” and the manufacturer’s protocol (RNeasy Mini Handbook 10/2013). The resulting DNA digestion was performed by using a Turbo DNA-free kit (Thermo Fisher Scientific, Waltham, MA, USA) according to the manufacturer’s recommendation. Transcription to cDNA was performed using 1 µg RNA and the iScript cDNA Synthesis Kit (Bio-Rad, Hercules, CA, USA) by following the manufacturer’s protocol. For each approach, the reverse transcriptase negative control was applied to detect putative DNA contamination. Synthesized cDNA was analyzed by PCR using specific primers for *rpoS* and *fliC* (Table 1). PCR was performed in a total volume of 25 μL containing 0.625 U *Taq* DNA polymerase (New England Biolabs Inc. (NEB), Ipswich, SD, USA), 200 μM dNTPs (NEB, USA), 0.2 μM of each primer (Eurofins Genomics GmbH, Ebersberg, Germany), 1× Standard *Taq* Reaction Buffer (NEB, USA), and 2 μL of the synthesized cDNA. The PCR reaction was performed with the following program: after the initial denaturation for 10 min at 95 °C, 35 cycles were carried out with each 60 s at 95 °C, 30 s at 52 °C (*rpoS*)/ 46 °C (*fliC*) and 70 s at 68 °C. After the final elongation at 68 °C for 10 min, the PCR products were cooled down to 8 °C. Correct amplification was confirmed by agarose gel (1% *w*/*v*) electrophoresis.

### 4.7. Soil Microenvironment Inoculation Experiments

The survival of *E. coli* O104:H4 C227/11Φcu in different soil types was investigated according to previously published persistence data with different modifications [39]. Two soil types were used, diluvial sand (DS) and alluvial loam (AL), which were kindly provided by Dr. Rita Grosch (Leibniz Institute of Vegetable and Ornamental Crops, Großbeeren, Germany). Both soil types were characterized by Schreiter et al. [35] and were already used in previous studies [36,37,38,39]. The soil inoculation experiments were performed in a microenvironment, consisting of 25 g soil, filled into 100 mL sterile cups (Sarstedt). Subsequently, the soil was adjusted to 50% of its maximum water holding capacity [35] using 10 mM MgCl_2_, and the lids were closed. For cattle manure supplementation, the amount was calculated regarding the maximum allowed amount of nitrogen (170 kg N/ha) per year for agricultural practices. In addition, the depth of incorporation into the soil was assumed to be 10 cm. The cattle manure with corresponding information concerning N-content was kindly provided from the Location Meiereihof with Kleinhohenheim, University of Hohenheim. These data are shown in Supplementary Table S1. For inoculation of the soil samples, bacterial strains were grown overnight in LB medium at 37 °C and 180 rpm. If required, LB medium was supplemented with chloramphenicol to a final concentration of 25 μg/mL. Afterwards, the cells were harvested by centrifugation at 5000× *g* for 5 min and resuspended in the same volume of 10 mM MgCl_2_. The OD_600nm_ of this suspension was measured, and the soil sample was mixed with the bacterial suspension to a final inoculum of 10^8^ colony forming units/g soil dry weight (cfu/g dw). All stated cfu counts (cfu/g soil) were always normalized to the dry weight of soil (cfu/g dw). As a control, the soil was mixed with 10 mM MgCl_2_. All samples were incubated at 4 °C or 22 °C for up to 12 weeks and analyzed directly after inoculation (day 0) and after 4, 8 and 12 weeks. Soil samples inoculated with C227/11Φcu were even incubated for up to 20 weeks. To recover *E. coli* cells from the soil, 1 g of soil (dw) was transferred to 50 mL conical tubes and suspended in 9 mL 0.9% NaCl (*w*/*v*) by vortexing for 1 min. Appropriate serial decimal dilutions were plated on TBX chromogenic agar (Carl Roth GmbH + Co. KG, Karlsruhe, Germany). If required, the agar was supplemented with 25 μg/mL chloramphenicol. The viable counts per gram of soil were calculated after incubation at 37 °C overnight. Each treatment was represented by triplicates in three independent replicates.

### 4.8. Statistical Analysis

Data were analyzed using the Brown–Forsythe test for variance homogeneity, followed by Welch’s one-way analysis of variance (ANOVA) with α = 0.05. For a better description of the survival curve of C227/11Φcu in AL and DS, the Weibull model was applied using GInaFiT [87] and the following formulation:

log_10_*N/N_0_* = −(*t*/*δ*)*^p^* (1)

with log_10_*N/N*_0_ as the log number of the relative population size (cfu/g soil), *t* as time in weeks, *p* as shape parameter and *δ* as scale parameter, which describes the time (weeks) for the first decimal reduction in cfu/g soil. Based on the resulting model parameters, the time required to reach the detection limit of 10^2^ cfu/g soil was calculated. The modeling of the other data sets was not possible since less than six observations were obtained during the experiments.

## 5. Conclusions

In conclusion, the results of the current study demonstrate that EHEC strain C227/11Φcu is able to survive for more than 12 weeks in soil microenvironments. The survival was improved at low temperatures and through the application of cattle manure. In addition, we identified the sigma factor RpoS as an important determinant for soil survival of C227/11Φcu. 

We established a method for soil inoculation and analyzed the survival of an stx2a-phage cured derivative of *E. coli* O104:H4 strain C227/11 in soil microenvironments. The strain was isolated during the large outbreak in 2011, and the transmission occurred by ingestion of sprouts. The EHEC/EAEC strain has high clinical importance and was therefore used for the experiments. The established method provided reliable results and is suitable to investigate the soil survival of further EHEC strains of different serotypes. Further experiments are needed to better understand the role of survival and persistence of human pathogens in agricultural soils.

## Figures and Tables

**Table 1:** *E. coli* strains and plasmids used in this study.

Strain or Plasmid	* Characteristics	Origin
Strains		
*E. coli* DH5α	*tonA lacZ*ΔM15 *endA1 recA1 thi-1 supE44 phoA gyrA96 hsdR17* Δ(*lacZYA-argF*)*U169 relA1*	Invitrogen
*E. coli* O104:H4 strain C227/11Φcu	Serotype O104:H4, cured from the *stx*_2a_-phage	[33]
*E. coli* O104:H4 strain C227/11Φcu/Δ*rpoS*	Deletion of *rpoS*	this study
*E. coli* O104:H4 strain C227/11Φcu/Δ*rpoS*/pFJ02	Deletion of *rpoS,* complemented, cam^R^	this study
*E. coli* O104:H4 strain C227/11Φcu/Δ*fliC*	Deletion of *fliC*	this study
*E. coli* O104:H4 strain C227/11Φcu/Δ*fliC*/pFJ03	Deletion of *fliC*, complemented, cam^R^	this study
Plasmids		
pKEC1.5	Derivative of plasmid pKD46, amp^R^ replaced by cam^R^	[48]
pKD4	Carries kan^R^ flanked by FRT sites	[49]
pCP20	Encoding for FLP recombinase, temperature-sensitive, cam^R^/amp^R^	[49]
pBR322	Cloning vector, pMB1 origin of replication, amp^R^, tet ^R^	[50]
pFJ01	pBR322 origin of replication, cam^R^ instead of amp^R^	this study
pFJ02	pBR322 origin of replication, cam^R^ instead of amp^R^, *rpoS* gene from *E. coli* O104:H4 C227/11Φcu	this study
pFJ03	pBR322 origin of replication, cam^R^ instead of amp^R^, *fliC* gene from *E. coli* O104:H4 C227/11Φcu	this study

* amp^R^ = ampicillin resistance. cam^R^ = chloramphenicol resistance. tet^R^ = tetracycline resistance. kan^R^ = kanamycin resistance.

**Table 2:** Oligonucleotide primers used in this study.

Name	* Sequence (5′-3′)	Function	Reference
P-*cat* PvuI for	ATA*CGATCG*AGCGCTGATGTCCGGC	Exchange of resistance	[37]
*cat* PvuI rev	ATA*CGATCG*TTACGCCCCGCCCTGCCA	Exchange of resistance	[37]
*fliC*del-O104-for	**AGCCCAATACTTAAACCGTAGACTTGAAAACAGGAAAATG**gcgattgtgtaggctggagc	Mutagenesis	This study
*fliC*del-O104-rev	**GCAGAAAAAACCCCGCCGGTAGCGGGGTCAGGCAGGTTAA**catggtccatatgaatatcctcc	Mutagenesis	This study
*rpoS*del-O104-for	**TTGAATGTTCCGTCAAGGGATCACGGGTAGGAGCCACCTT**gcgattgtgtaggctggagc	Mutagenesis	This study
*rpoS*del-O104-rev	**CCAGCCTCGCTTGAGACTGGCCTTTCTGACAGATGCTTAC**catggtccatatgaatatcctcc	Mutagenesis	This study
*fliC*-O104-for	CCCAAGCGTTGAAATACTAGCCA	Confirmation of mutagenesis	This study
*fliC*-O104-rev	CTTCAGCGGTATAGAGTGAATTCA	Confirmation of mutagenesis	This study
*rpoS*-O104-for	CTGCGTTATTTGCCGCAGCG	Confirmation of mutagenesis	This study
*rpoS*-O104-rev	GTGCGCAAGATGATGAACGCAT	Confirmation of mutagenesis	This study
*fliC*-HindIII-O104-for	CGC*AAGCTT*ATGGCACAAGTCATTAATA	Complementation	This study
*fliC*-BamHI-O104-rev	TAT*GGATCC*TTAGCCTTGTAACAGAGA	Complementation	This study
*rpoS*-HindIII-O104-for	CCC*AAGCTT*ATGAGTCAGAATACGCTGAAA	Complementation	This study
*rpoS*-BamHI-O104-rev	AAT*GGATCC*TTACTCGCGGAACAGCG	Complementation	This study

* The homologous regions for recombineering are highlighted in bold, and letters in italics indicate restriction sites.

**Table 3:** Weibull parameters of C227/11Φcu survival in AL and DS at 22 °C and 4 °C determined with GInaFiT. *δ* (first decimal reduction in weeks), *p* (shape parameter), *R*^2^ (regression coefficient), the time for 4 log reduction and the time to reach detection limit in weeks are shown.

C227/11Φcu in	*δ*	*p*	*R* ^2^	Time for 4 Log Reduction (Weeks)	Time to Reach Detection Limit of 10^2^ cfu/g Soil (Weeks)
DS 22 °C	1.73	0.83	0.9642	±9.2 weeks	±13.3
AL 22 °C	1.15	0.74	0.9678	±7.6 weeks	±12.3
DS 4 °C	2.89	0.88	0.9639	±14 weeks	-
AL 4 °C	11.11	1.87	0.9657	-	-

## Data Availability

The datasets generated and/or analyzed during the current study are available from the corresponding author on reasonable request.

## Supplementary Materials

The following are available online at https://www.mdpi.com/article/10.3390/pathogens10111443/s1, Figure S1: Agarose gel electrophoresis to confirm the incorporation of chloramphenicol resistance cassette to construct isogenic *rpoS* deletion mutant of C227/11Φcu, Figure S2: Agarose gel electrophoresis to confirm the removal of chloramphenicol resistance cassette for the construction of C227/11Φcu Δ*rpoS*, Figure S3: Agarose gel electrophoresis to confirm the construction of the *fliC* deletion mutant of C227/11Φcu by the incorporation of camR (A) and the removal of the antibiotic resistance cassette (B), Figure S4: Agarose gel electrophoresis of cDNA of *rpoS* (A) and *fliC* (B) expressed by pFJ02 and pFJ03, respectively, Figure S5: Use of the Weibull model to describe the survival curves of *E. coli* O104:H4 C227/11Φcu in (A) DS at 22 °C, (B) AL at 22 °C, (C) DS at 4 °C, and (D) AL at 4 °C. Figure S6: Analysis of soil survival of *E. coli* O104:H4 C227/11Φcu Δ*rpoS*/pFJ02 (A) and C227/11Φcu Δ*fliC*/pFJ03 (B) depending on soil type and temperature, Table S1: Cattle manure composition.
