# Supplementary material for: Survival of Enterohemorrhagic Escherichia coli O104:H4 Strain C227/11Φcu in Agricultural Soils Depends on rpoS and Environmental Factors
Source: Pathogens. 2021 Nov 5;10(11):1443. doi: 10.3390/pathogens10111443 (PMC8620961; doi:10.3390/pathogens10111443)
Supplement: Supplementary file 1 [file pathogens-10-01443-s001.zip › pathogens-1433931-supplementary.pdf]

Table S1. Cattle manure composition.

Density [kg/L]	Total-N [%]	NH_4-N [%]	Total Phosphate (P_2O_5) [%]	Total Potassium (K_2O) [%]
1.028	0.212	0.115	0.0961	0.179

Table S1 shows the composition of the cattle manure used for the experiments. The samples with the corresponding information about manure composition were kindly provided from the Location Meiereihof with Kleinhohenheim, University of Hohenheim.

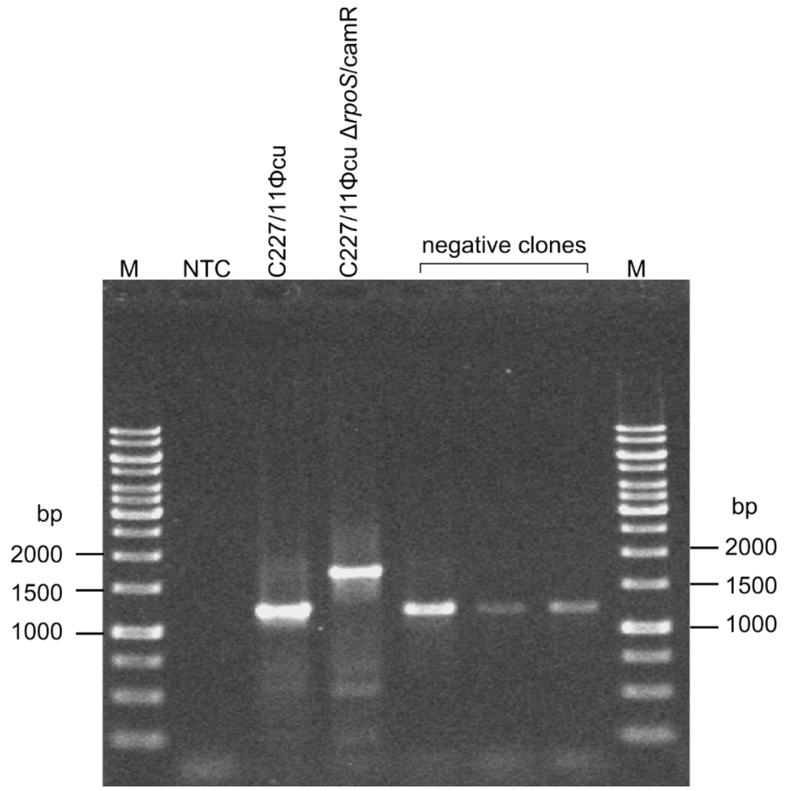

Figure S1. Agarose gel electrophoresis to confirm the incorporation of chloramphenicol resistance cassette to construct isogenic *rpoS* deletion mutant of C227/11Φcu.

The sample with DNA from C227/11Φcu as a template shows an amplicon with a size of ~1200bp. The same band size was obtained for negative clones. The sample showing a band at ~1700 bp indicates the incorporation of the chloramphenicol resistance cassette (cam^R). Marker (1 kb DNA ladder, GeneRuler, Thermo Scientific, USA) and no template control (NTC) in PCR are shown.

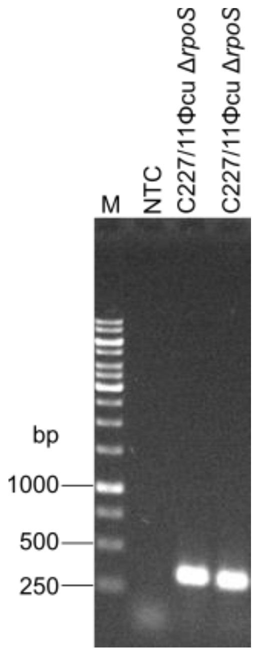

Figure S2. Agarose gel electrophoresis to confirm the removal of chloramphenicol resistance cassette for the construction of C227/11Φcu Δ*rpoS.*

In Figure S2, the removal of chloramphenicol resistance cassette is shown, which is indicated with bands at ~400 bp. The construction of C227/11Φcu Δ*rpoS* was successful, which was further confirmed by sequence analysis. The figure also contains the marker (1 kb DNA ladder, GeneRuler, Thermo Scientific, USA) and no template control (NTC).

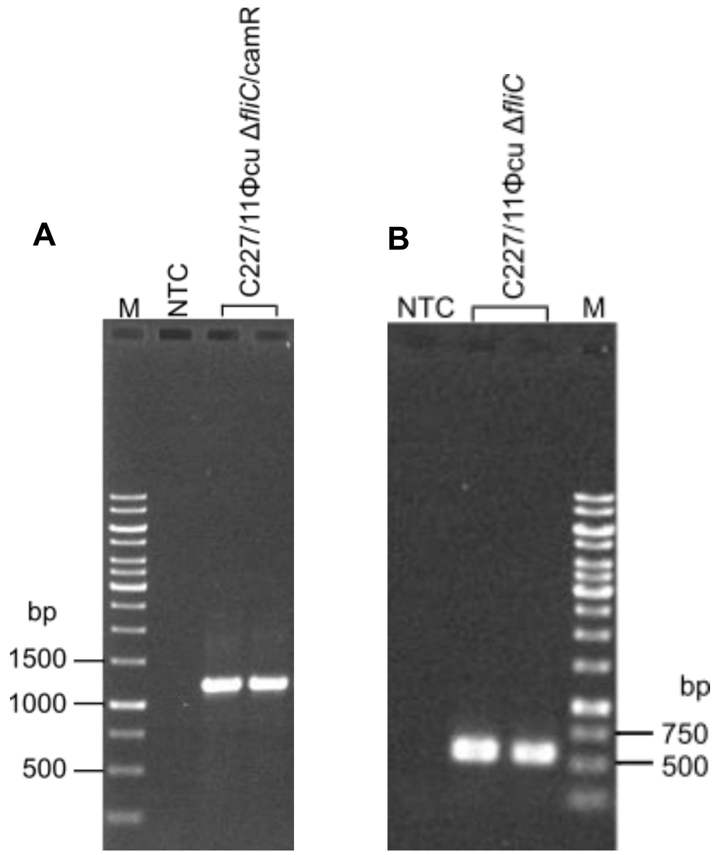

Figure S3. Agarose gel electrophoresis to confirm the construction of *fliC* deletion mutant of C227/11Φcu by incorporation of cam^R (**A**) and the removal of the antibiotic resistance cassette (**B**).

In Figure S3 A, the samples show an amplicon after PCR using DNA from two clones as a template. The band sizes of ~1200 bp indicate the incorporation of the chloramphenicol resistance cassette, and therefore, the successful construction of C227/11Φcu Δ*rpoS*/cam^R. In Figure S3 B, the removal of chloramphenicol resistance cassette is shown, which is indicated with bands at ~550 bp. The construction of C227/11Φcu Δ*fliC* was successful, which was further confirmed by sequence analysis. Both the agarose gel electrophoresis marker (1 kb DNA ladder, GeneRuler, Thermo Scientific, USA) and the no template control (NTC) were used.

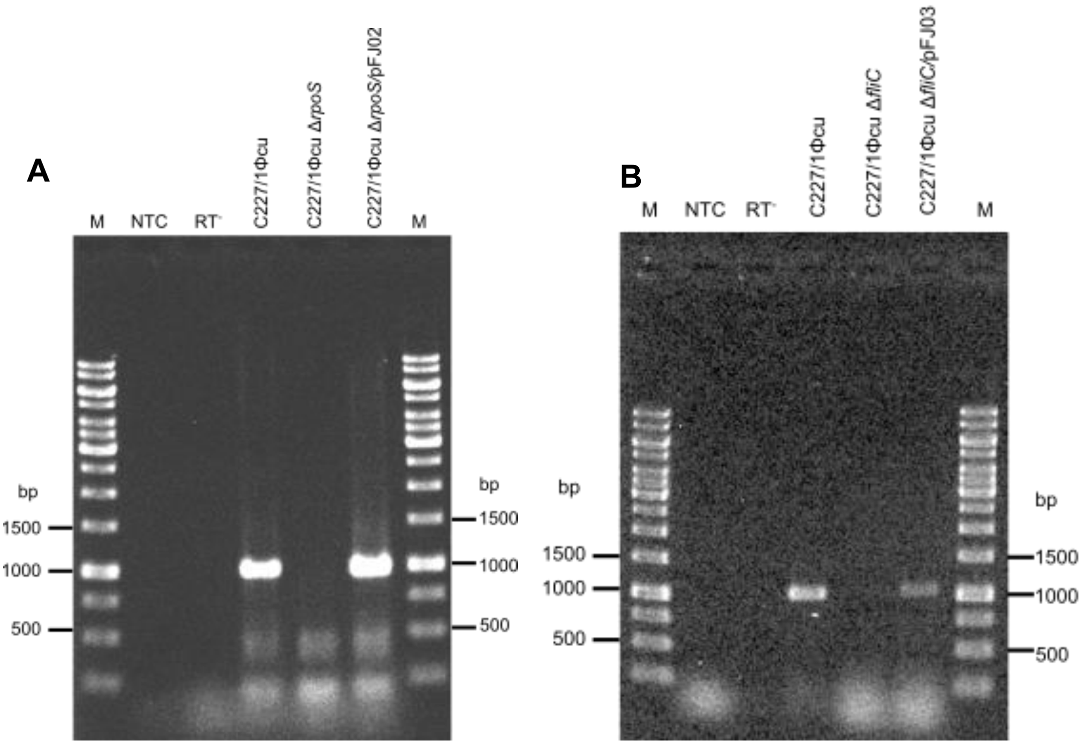

Figure S4. Agarose gel electrophoresis of cDNA of *rpoS* (**A**) and *fliC* (**B**) expressed by pFJ02 and pFJ03, respectively.

Samples in Figure S4 (A) show amplicons after PCR with cDNA samples of C227/11Φcu, C227/11Φcu Δ*rpoS* and complemented strain C227/11Φcu Δ*rpoS*/pFJ02. Figure S4 (B) shows amplicons after PCR with cDNA samples of C227/11Φcu, C227/11Φcu Δ*fliC* and complemented strain C227/11Φcu Δ*fliC*/pFJ03. The reverse transcriptase negative control (RT$^-$) of the isogenic deletion strains are shown in the respective figures. In addition, marker (1 kb DNA ladder, GeneRuler, Thermo Scientific, USA) and no template control (NTC) of the PCR are shown.

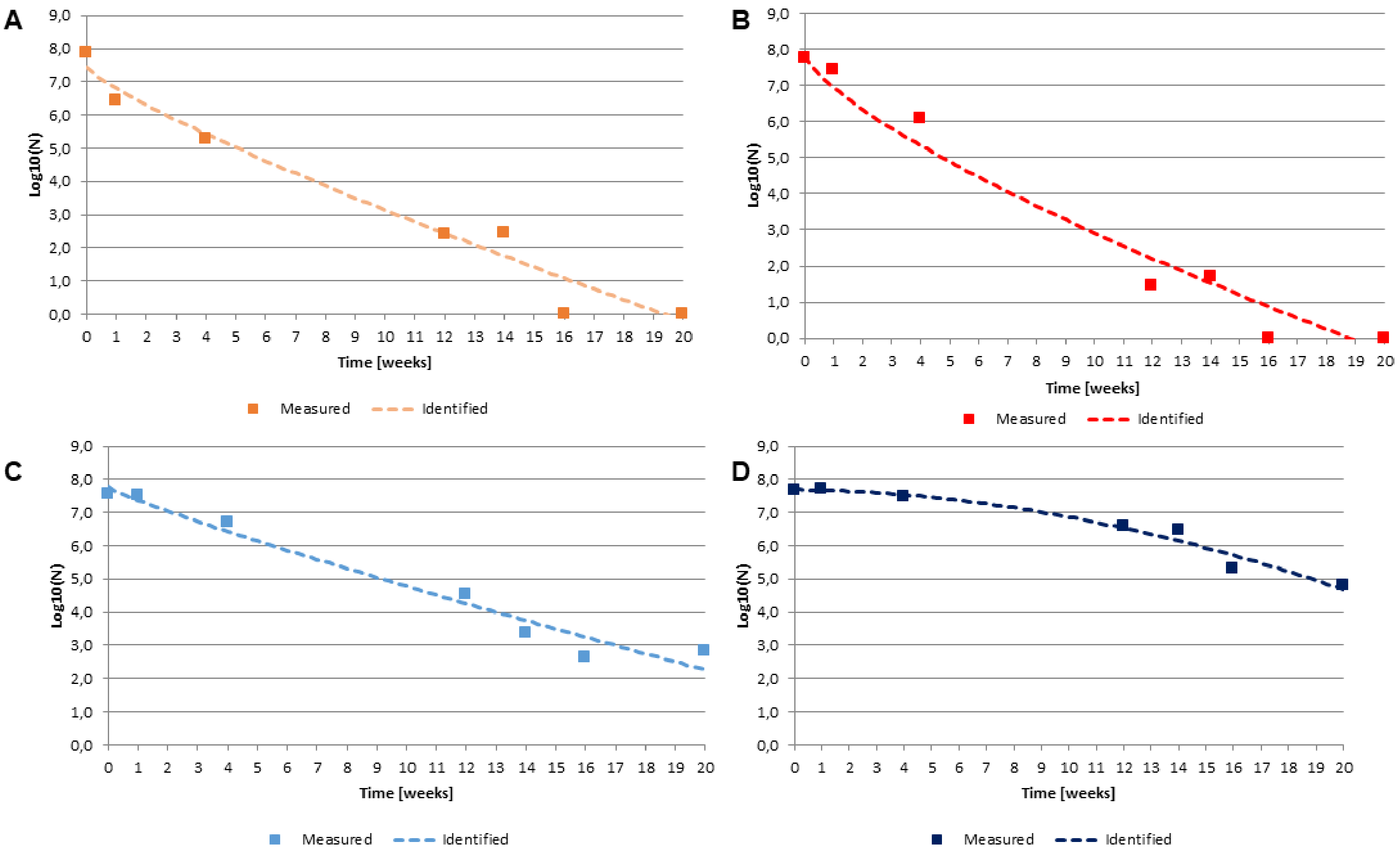

Figure S5. Use of Weibull model to describe the survival curves of *E. coli* O104:H4 C227/11Φcu in (A) DS at 22°C (B) AL at 22°C (C) DS, at 4°C and (D) AL at 4°C.

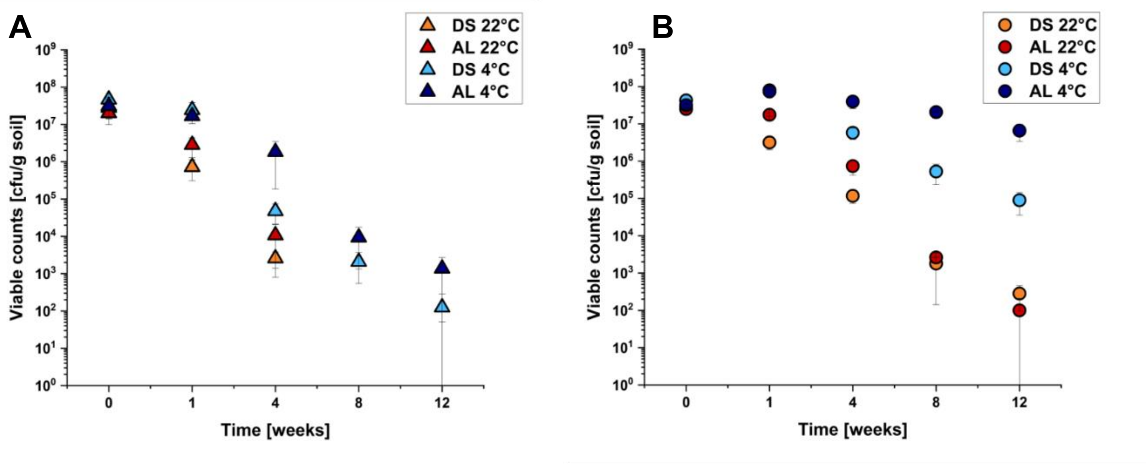

Figure S6. Analysis of soil survival of *E. coli* O104:H4 C227/11Φcu Δ*rpoS*/pFJ02 (A) and C227/11Φcu Δ*fliC*/pFJ03 (B) depending on soil type and temperature (as indicated). The soil was inoculated with 10^8 cfu/g soil and incubated for 12 weeks. Data are means ± standard errors of the experiments performed in triplicates.
